# Analysis of Vaccenic and Elaidic acid in foods using a silver ion cartridge applied to GC × GC-TOFMS

**DOI:** 10.3389/fnut.2023.1320550

**Published:** 2024-01-08

**Authors:** Miyoung Yoo, Hyun Jeong Lee, Kwang-Won Lee, Dongwon Seo

**Affiliations:** ^1^Food Standard Research Center, Korea Food Research Institute, Wanju-gun, Jeollabuk-do, Republic of Korea; ^2^Department of Biotechnology, Korea University, Seongbuk-gu, Seoul, Republic of Korea; ^3^Food Analysis Research Center, Korea Food Research Institute, Wanju-gun, Jeollabuk-do, Republic of Korea

**Keywords:** Vaccenic acid, elaidic acid, silver ion cartridge, GC × GC-TOFMS, trans fatty acids

## Abstract

**Background:**

*Trans* fatty acids (TFAs) are unsaturated fatty acids, with vaccenic acid (VA) and elaidic acid (EA) being the major constituents. While VA has been associated with beneficial effects on health and anti-cancer properties, EA is found in hardened vegetable oils and is linked to an increased risk of cardiovascular diseases. Therefore, this study aimed to develop a novel method for the quantitative measurement of VA and EA, aiming to accurately analyze individual TFA and apply it for the assessment of products containing TFAs.

**Methods:**

The ratio of VA to EA (V/E ratio) was evaluated using a silver ion cartridge (*SIC*) solid phase extraction method removing *cis*-fatty acids (*cis*-FAs). Additionally, comparative analysis of the V/E ratio was conducted by the two methods (*SIC* treatment and untreated) using comprehensive two-dimensional gas chromatography combined with time-of-flight mass spectrometry (GC × GC-TOFMS).

**Results:**

The removal efficiency of *cis*-FAs was greater than 97.8%. However, the total TFA contents were not so different from *SIC* treatment. Moreover, this approach not only allowed for a more precise determination of the V/E ratio but also revealed a significant distinction between natural *trans* fatty acids (N-TFAs) and hydrogenated *trans* fatty acids (H-TFAs).

**Conclusion:**

Therefore, the *SIC* coupled to the GC × GC-TOFMS presented in this study could be applied to discriminate N-TFA and H-TFA contents in dairy and fatty foods.

## Introduction

*Trans* fatty acids (TFAs) represent a category of unsaturated fatty acids characterized by at least one double bond in a *trans* configuration (1). These TFAs encompass various fatty acids, including vaccenic acid (11 t-C18:1, VA), elaidic acid (9 t-C18:1, EA), and other substances. Both VA and EA are recognized as major fatty acids in C18:1 TFAs. (2–4). Despite their similar chemical structures, the position of the carbon–carbon double bond plays role in determining their impact on human health (5, 6). EA, prevalent in partially hydrogenated vegetable oils, is linked to an elevated risk of cardiovascular disease, displaying a negative correlation with plasma HDL-cholesterol levels and a positive correlation with plasma LDL-cholesterol levels (7–9). Conversely, studies suggest that VA may have beneficial health effects, including potential anti-cancer effects properties (10–13). In hydrogenated *trans* fatty acids (H-TFAs), EA serves as a a primary source, forming a diverse range of TFAs mixtures (14). Natural *trans* fatty acids (N-TFAs) found in the human diet, particularly in animal fat sources like butter and cheese, predominantly consist of VA, which constituting over 60% of total TFAs (14).

Previous studies (14) have examined the levels of VA and EA in foods, proposing the ratio of VA to EA as a distinguishing factor between N-TFAs and H-TFAs. However, accurate estimation of this ratio requires the separation of *trans*- and *cis*-FAs. The presence of a *cis* bond induces a bend or flexion in the fatty acid chain, while a *trans* bond results in a structure resembling that of a saturated fatty acid due to the straightening of the chain (15). Clear separation, identification and quantification of TFAs pose challenges due to overlapping isomers of *trans*- and *cis*-FAs (16–18).

Capillary zone electrophoresis (CE) emerges as a method capable of identifying EA and VA with minimal organic solvents and reagents, without requiring specific columns (19). However, the presence of matrix components, especially proteins, in the sample can lead to decreased separation efficiency due to their adsorption on the hydrophilic surface of the silica capillary (20, 21). Recent students (18, 22) propose the use of silver ions anchored onto a strong cation exchange for solid phase extraction (SPE) to remove isomers of *cis*-FAs from commonly consumed foods. Kramer et al. (17) analyzed eighty-seven fatty acids in milk samples using a silver ion cartridge (*SIC*) SPE method. They recommended gas chromatography combined with electron ionization mass spectrometry (GC/EI-MS), comprehensive two-dimensional gas chromatography (GC × GC) combined with a flame ionization detector (FID), and silver ion high performance liquid chromatography interfaced with atmospheric pressure photoionization mass spectrometry (HPLC/APPI-MS) for accurate determination of individual TFAs.

While HPLC/MS detection proved to be a rapid and reproducible method for nine C18:1 fatty acid methyl ester isomers, time-of-flight mass spectrometry (TOFMS) offered advantages in resolution and sensitivity without sacrificing full mass spectra information (23). Additionally, GC × GC experiments allowed for versatile separation of complex mixtures in a single run, demonstrating reproducibility in retention time in both dimensions (24).

However, to the best of our knowledge, there is currently no method for identifying EA and VA in various foods using the combination of GC × GC and TOFMS after *SIC* pretreatment, considering the advantages of different analytical techniques. Therefore, the combination of GC × GC and TOFMS could be suggested as one of the most efficient analytical methods for the isolation and identification of individual TFA. In this study, our primary objective was to investigate an extraction method for removing *cis*-fatty acids, enabling a more precise analysis of individual TFAs. Furthermore, we aimed to separate and quantify EA and VA to assess the distribution of TFA isomers in various *trans*-fat-containing foods, including margarine, butter, cheese, and milk.

## Materials and methods

### Chemicals

Individual reference fatty acid methyl ester (FAME) standards, including of *trans*-9-elaidic methyl ester (9 t-C18:1, EA) and *trans*-11-vaccenic methyl ester (11 t-C18:1, VA) were purchased from Nu-Chek Prep Inc. (Elysian, MN, USA). All solvents and reagents utilized in this study were of analytical grade. A 14% boron-trifluoride methanol solution (BF_3_), sodium hydroxide, and sodium chloride were obtained from Sigma-Aldrich (St. Louis, MO, USA). Additionally, chloroform, normal hexane (HPLC grade, 95%), and methanol were purchased from J. T. Baker (Philipsburg, NJ, USA).

### Sample preparation

Representative TFA-containing food samples commonly found in the general market were selected for analysis, chosen specifically for their high TFA content (25). A total 30 samples, including margarine, butter, cheese, and ice cream, were obtained from a local grocery store in South Korea. Among these, there are 3 samples of margarine, 3 of butter, 7 of cheese, and 17 of ice cream comprising various product types.

Fat from these samples was extracted using a mixture of chloroform and methanol (2:1, v/v) and subsequently evaporated to dryness (26, 27). In the extraction process, 20 mg of fat was placed into a vial with 2 mL of 0.5 M methanolic sodium hydroxide and capped. The vial was then heated at 100°C for 5 min, followed by cooling at 25°C. Subsequently, 2 mL of BF3 reagent was added, and the mixture was heated at 100°C for an additional 5 min. To this, 2 mL of isooctane and saturated sodium chloride solution were added, followed by vortexing for 1 min. The isooctane layer was transferred to a separate vial.

To eliminate *cis*-FAs from the test solutions, 1 mL of the test solution in isooctane was loaded onto the preconditioned *SIC* (6 mL, Supelco, Bellefonte, USA) after pre-conditioning with 4 mL acetone and 4 mL of n-hexane. For *SIC* elution, solutions were eluted with hexane and acetone ratios of 99:1, 94:4, and 90:10. Four mL of n-hexane/acetone (96:4) was used and collected in a 12 mL vial. The solution in the vial was then evaporated to dryness using nitrogen gas. The residues in the vial were dissolved with 2 mL of isooctane for subsequent GC × GC-TOFMS analysis.

### Analysis conditions of GC-FID, GC × GC-FID, and GC × GC-TOFMS

The GC-FID was operated with an Agilent 6,890 N GC (Agilent Technologies, Santa Clara, CA, USA) equipped with a flame ionization detector (FID). The injector temperature was set at 230°C, the detector temperature was 250°C, and the column oven temperature was increased from 120°C to 230°C at a rate of 5°C per min. A SP-2560 column (100 m × 0.25 mm i.d., 0.25 μm film thickness, Supelco, CA, USA) was utilized, and helium served as the carrier gas with a flow rate maintained at 1.5 mL/min.

For GC × GC-FID, a LECO Corporation Pegasus 4D instrument with an Agilent 6,890 N GC was employed. The GC × GC analysis involved a primary column SP-2560 (100 m × 0.25 mm i.d., 0.25 μm film thickness, Supelco, CA, USA) and a secondary column RTX-5 (1.5 m × 0.18 mm i.d., 0.18 μm film thickness, Restek, Bellefonte, PA, USA). The main oven temperature was initially held at 45°C for 4 min, increased to 175°C at a rate of 13°C/min and held for 27 min. Subsequently, it was raised to 215°C at a rate of 4°C/min and held for 10 min. The secondary oven was set 20°C higher than the main oven temperature. The modulator temperature offset was 40°C, the second dimension separation time was 5 s, the cool time between stages was 1.5 s and the hot pulse time was 1.0 s.

For GC × GC-TOFMS, the operating conditions mirrored those of GC × GC-FID. GC × GC-TOFMS was run at an acquisition rate of 100 spectra/s. The transfer line and the ion source temperature were at 220°C. The electron energy was 70 eV, and mass spectra were collected in the m/z range of 35–500. Spectra were identified using the NIST Mass Spectral Search Program through Chroma TOF-GC software for PEGASUS 4D of LECO Corp.

### Validation of GC × GC-TOFMS method

The GC × GC-TOFMS method underwent validation for parameters including linearity, sensitivity, and precision. Linearity of the calibration curves was examined for each EA and VA standard at varing concentrations within the range of 1–100 μg/mL, with all evaluations conducted in triplicate. Limits of detection (LOD) and limits of quantification (LOQ) for EA and VA standards were experimentally determined through serial dilutions until signal-to-noise ratios of 3 and 10 were reached, respectively.

Precision was assessed by multiple injections of several standard curve levels (ranging from 1–100 μg/mL) intra-day for repeatability and inter-day for intermediate precision. Repeatability represents the variability of independent results obtained by analyzing the sample six times, was measured by calculating the relative standard deviation (RSD) values for all the collected data. To evaluate the accuracy of the analytical method, recovery tests were conducted.

### Statistical analysis

The experiment was conducted in triplicate, and the results are expressed as the mean ± standard deviation. Statistical analysis was performed using the SPSS 13.0 software for Window (LEAD TOOLS, LEAD Technologies, Inc., 2004).

## Results and discussion

### Condition on *SIC* fractionation

Table 1 shows the distribution of fatty acids eluted by *SIC* with different ratios of acetone in hexane (H:A), as analyzed using GC-FID. Total methylated lipids were applied to the *SIC*, and the H:A ratio was increased to 99:1, 96:4 and 90:10, respectively, with elution carried out using 4 mL volumes of mixed solvent. In the untreated *SIC*, C18:0, C18:1-*trans*, and C18:1-*cis* were found to be 15.7 g/100 g, 17.3 g/100 g, and 26.5 g/100 g, respectively. Upon *SIC* treatment, C18:0 and C18:1-*cis* were eluted in fractions 1 and 3, while in fraction 2, 16.9 g/100 g of C18:1-*trans* was eluted with 97.7% recovery. Consequently, it was determined that the H:A 96:4 solution was the most suitable solution for separating individual TFAs.

**Table 1:** Fatty acid fractionation of margarine using a silver ion cartridge (*SIC*) solid phase extraction method.

Margarine (*n* = 3)	Fatty acid contents (g/100 g)		
	C18:0	C18:1-*trans*	C18:1-*cis*
Untreated	15.7 ± 0.4	17.3 ± 0.5	26.5 ± 1.2
*SIC* treated			
Fr. 1, H:A^*^ (99:1)	15.5 ± 0.4	0.1 ± 0.0	N.D.
Fr. 2, H:A (96:4)	N.D.	16.9 ± 0.4	1.5 ± 0.1
Fr. 3, H:A (90:10)	N.D.	N.D.	24.9 ± 1.1

*H:A = hexane:acetone.

### *Cis* fatty acids removal and C18:1 TFAs separation

**Figure 1:**
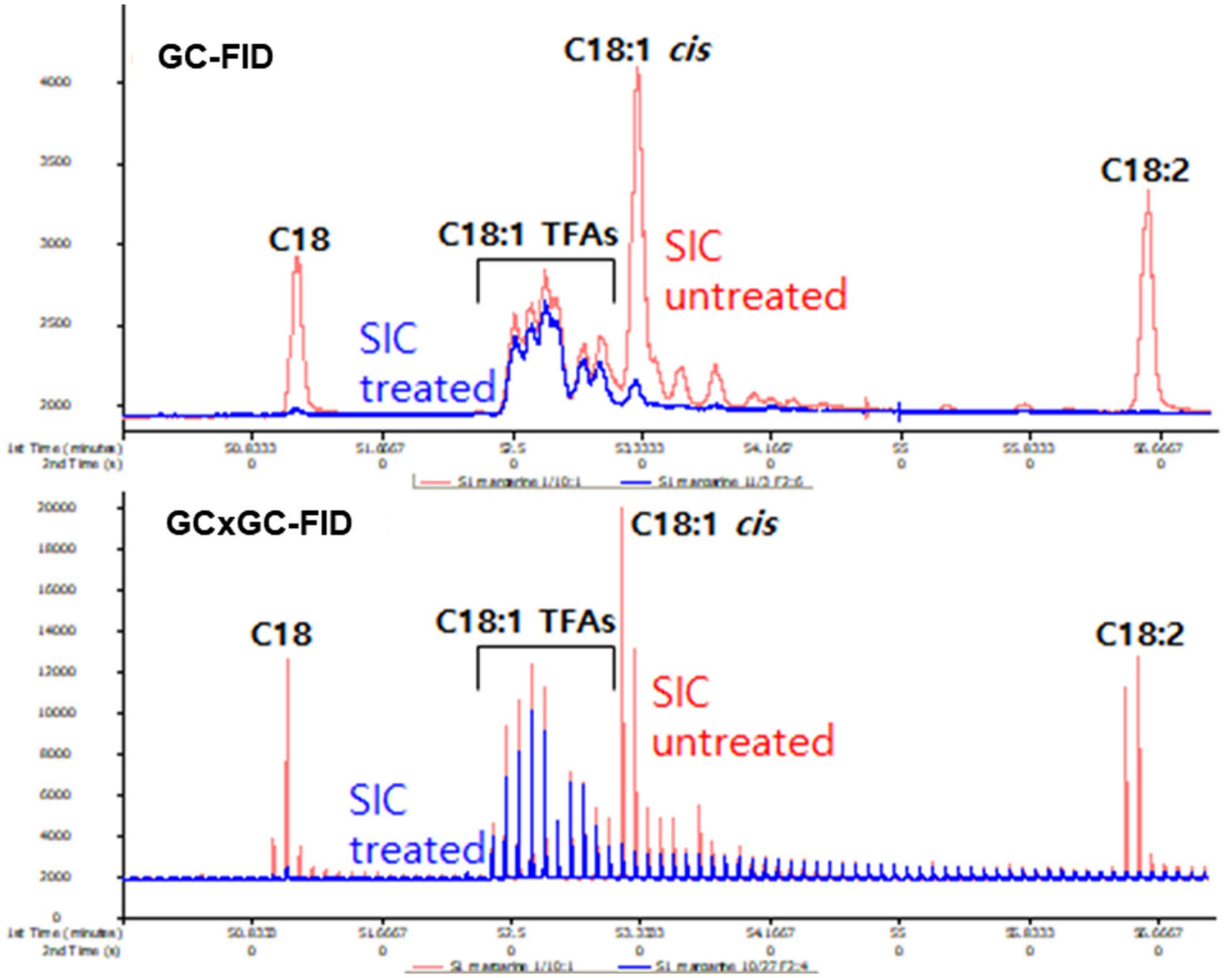
The effect of *SIC* treatment for removing *cis*-FA by GC-FID and GC × GC-FID.

The effectiveness of *cis*-fat removal through *SIC* treatment was verified through GC-FID and GC × GC-FID chromatogram (Figure 1). Except for C18:1 TFA, *SIC* treatment demonstrated efficient removal of other fatty acids, such as C18:0, C18:1-*cis* and C18:2.

Despite advancements in capillary column resolution, the development of a single column for separating individual TFA isomers remains elusive. A recent breakthrough involves a two-dimension technique that utilizes GC coupled with a modulator (GC × GC) to physically separate the elution of the first column, enabling the analysis of each fraction in the secondary column. Ongoing research is actively exploring ways to effectively separate and analyze isomers with different double bond positions using GC × GC (14, 24, 28). Using these GC × GC techniques, TFA isomeric peaks, which were indistinguishable in the GC-FID chromatogram (Figure 1A), were successfully resolved into individual TFA isomers in the GC × GC-FID representation (Figure 1B).

### Separation of EA and VA by GC × GC-TOFMS

**Figure 2:**
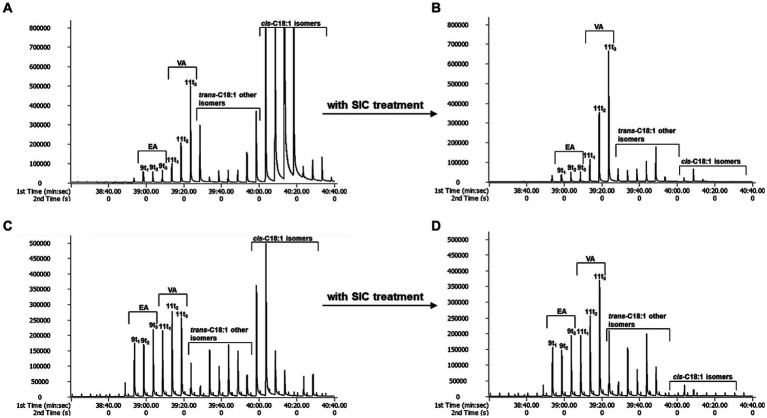
The effect of *SIC* treatment by GC × GC-TOFMS. **(A)**: butter, **(B)**: butter with *SIC* treatment, **(C)**: margarine, and **(D)**: margarine with *SIC* treatment.

When TFAs were analyzed using GC × GC-TOFMS, they were separated into individual isomers to the greatest extent than GC × GC-FID analysis (Figure 1). *Cis*-FAs were eluted after the TFAs group in chromatograms (A and C in Figure 2), and clear elimination of them was observed (B and D in Figure 2).

**Figure 3:**
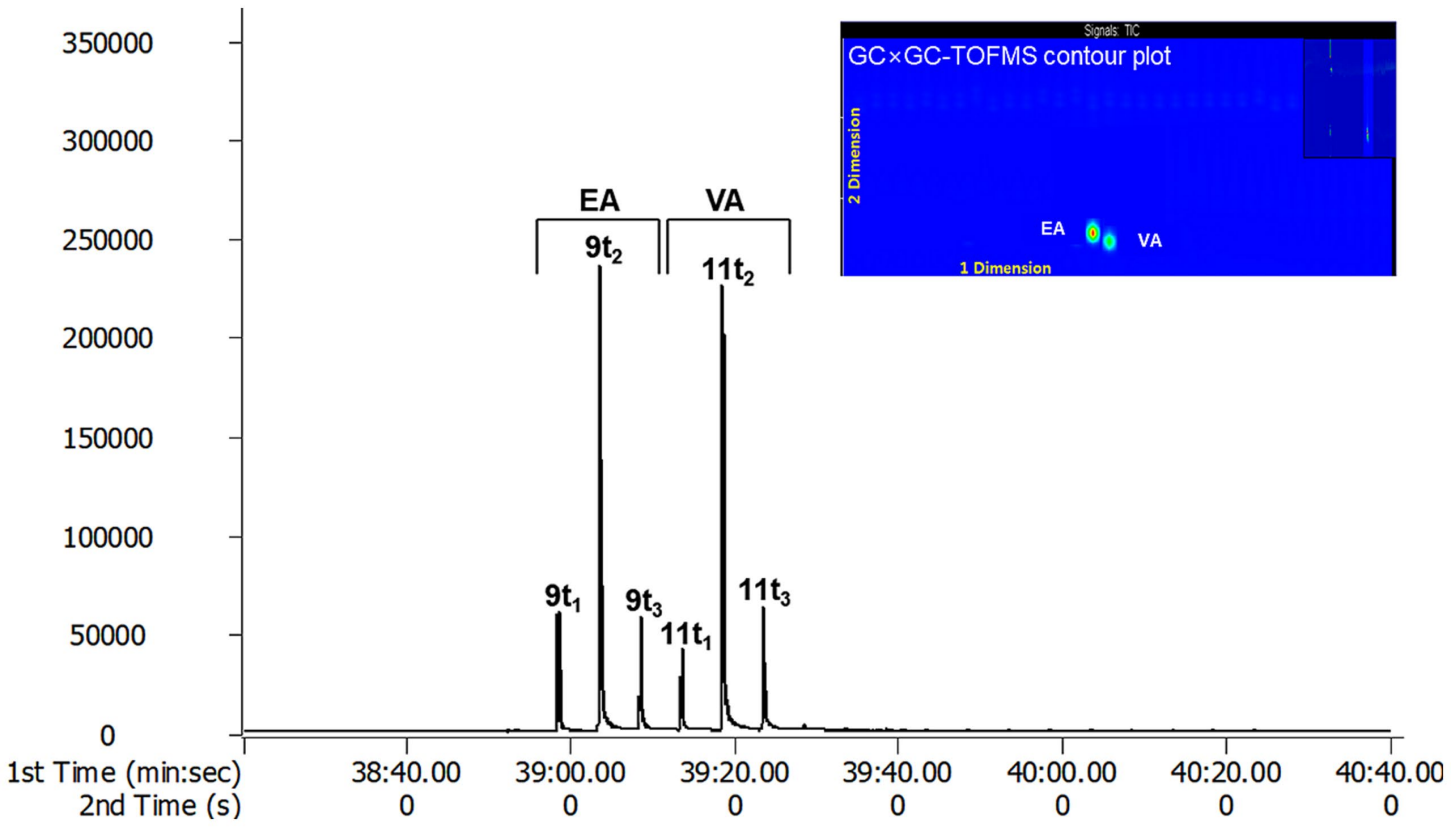
The standard chromatogram and contour plot of EA and VA by GC × GC-TOFMS.

The effective separation of individual TFA by TOFMS is attributed to the sufficiently high mass resolution. This allows for the separation of peaks even if the masses are the same, dramatically improving the identification of specific molecular fragments and reducing cross-sensitivity. Additionally, it can diminish background noise generated by disturbance ion signals or compensate for co-elution of non-target species in GC × GC (23). In conclusion, GC × GC-TOFMS offers advantages in resolution and sensitivity without sacrificing the overall mass spectral information of EA and VA. Through the analysis of EA and VA standards with GC × GC-TOFMS, EA was separated into peaks 9 t1, 9 t2 and 9 t3, and VA into 11 t1, 11 t2, and 11 t3 using a modulator (Figure 3). The 9 t2 and 11 t2 are primary peaks for EA and VA. 9 t1, and 9 t3 are subpeaks for EA, and 11 t1 and 11 t3 are subpeaks for VA, respectively. As depicted in A and C in Figure 2, the analysis of butter and margarine samples revealed that C18:1 TFAs comprised EA and VA. However, the properties of other isomers, excluding EA and VA, have not been precisely confirmed, necessitating further research.

### Method validation of EA and VA analysis using GC × GC-TOFMS with *SIC* treatment

Analytical validation holds principal importance, establishing scientific evidence that an analytical procedure produces reliable results (29). Analytical methods, crucial for ensuring product quality characteristics, gains validity through a proper validation process. This involves a formal, systematic, and well-documented assessment of the analytical method’s capability to provide accurate, reliable, and reproducible results.

To validate the proposed GCxGC-TOFMS method, various parameters, including linearity, LOD, LOQ, precision, and recovery, were considered and evaluated. Linearity and range standards were serially diluted to obtain five concentration levels. The correlation coefficients of the regression lines of each TFA standard were 0.9997 for EA and 0.9993 for VA. Method sensitivity was assessed by the LOD and LOQ, with EA LOD and LOQ at 2.20 and 4.38 μg/mL, respectively, and VA LOD and LOQ at 6.66 and 13.27 μg/mL, respectively (Table 2).

**Table 2:** Linearity, limits of detection (LOD), and limits of quantification (LOQ) of elaidic acid (EA) and vaccenic acid (VA).

Analytes	Linear range (μg/mL)	Correlation coefficient (R)	LOD (μg/mL)	LOQ (μg/mL)
EA	1.0–100.0	0.9997	2.20	6.66
VA	1.0–100.0	0.9993	4.38	13.27

Method precision was evaluated by repeatability and reproducibility. The intra- and inter-day precision were assessed using EA and VA results in the four samples, revealing RSD values for intra-day precision ranging from 1.8 to 8.9%, and inter-day precision ranging from 3.9 to 8.1%. The validation data demonstrated precision below 20% for all the tested samples, indicating excellent method performance across the entire calibration range (30). To analyze recovery rates, EA and VA were spiked into margarine, butter, cheese, and ice cream. The recovery rate of EA ranged from 91.1 to 98.4%, while VA ranged from 91.5 to 102.6%. Detailed results are summarized in Table 3.

**Table 3:** Method validation of precision and accuracy.

Analytes	Linear range (μg/mL)	Intra-assay RSD^a^ (%) (*n* = 3)	Inter-assay RSD (%) (*n* = 9)	Recovery (%)
Margarine	Elaidic acid	3.3	4.7	92.7 ± 5.5
	Vaccenic acid	4.2	4.5	97.2 ± 5.1
Butter	Elaidic acid	5.0	3.9	93.4 ± 5.0
	Vaccenic acid	8.9	7.0	100.1 ± 2.4
Cheese	Elaidic acid	8.6	5.2	91.1 ± 2.5
	Vaccenic acid	4.3	6.1	91.5 ± 3.1
Ice cream	Elaidic acid	1.8	4.1	98.4. ± 4.8
	Vaccenic acid	2.1	8.1	102.6 ± 3.8

a RSD: relative standard deviation.

### Effect of *SIC* treatment on VA and EA contents

Table 4 presents the results related to the total fat, TFAs, VA and EA contents of margarine, butter, cheeses, and ice creams. The total fat content varied from 8.0 g/100 g for ice cream to 99.8 g/100 g for margarine. TFA contents ranged from 1753.7 mg/100 g cheese (*SIC* treated) to 17314.3 mg/100 g margarine (*SIC* treated). In the case of margarine, TFAs decreased by 2.2% from 17314.3 mg/100 g to 16940.5 mg/100 g due to *SIC* treatment. With *SIC* treatment, similar reduction of 9.9 and 5.3% were observed in butter and cheese, both derived from natural milk. Ice cream, categorized into two groups based on nutrition facts (group 1 with added hydrogenated fat and group 2 with natural milk fat), showed a 3.8% decrease in TFAs in group 1 and a 9.0% decrease in group 2 with *SIC* treatment.

**Table 4:** Variation of total fat, *trans* fatty acids (TFAs), VA, and EA contents by *SIC* treatment.

Analytes	Total fat (g/100 g)	*SIC*	TFAs (mg/100 g)	VA (mg/100 g)	EA (mg/100 g)
Margarine (*n* = 3)	99.8	Untreated	17314.3 ± 714.2	5459.4 ± 141.1	5453.9 ± 175.5
		Treated	16940.5 ± 1055.6	5777.5 ± 298.2	5555.4 ± 253.2
Butter (*n* = 3)	90.5	Untreated	3164.0 ± 152.3	1263.1 ± 48.0	429.6 ± 15.4
		Treated	2879.1 ± 111.8	1912.3 ± 59.3	262.5 ± 8.1
Cheese (*n* = 7)	32.2	Untreated	1846.6 ± 152.7	820.4 ± 64.2	244.2 ± 14.3
		Treated	1753.7 ± 59.6	1046.5 ± 30.0	135.0 ± 3.4
Ice cream (*n* = 11) H^a^	8.0	Untreated	3522.5 ± 504.9	1394.6 ± 211.6	690.4 ± 115.4
		Treated	3202.1 ± 411.5	1665.9 ± 200.3	425.0 ± 54.4
Ice cream (*n* = 6) N^b^	13.9	Untreated	2837.8 ± 253.8	1407.0 ± 132.1	349.1 ± 21.1
		Treated	2511.1 ± 200.4	1525.0 ± 124.4	202.6 ± 16.6

a Added hydrogenated fat to milk fat.

b Only natural milk fat.

The analysis of VA and EA, major components of C18:1 TFAs, can be affected by *cis*-FAs due to overlapped elution. Despite the 163°C GC temperature regimen with *SIC* treatment facilitating the detection of 15 t-18:1, effective differentiation between the 6 t/7 t/8 t- to 11 t-18:1 isomers remained challenging (17). While GC–MS is an effective method for determining fatty acid structures, its application in the analysis of TFAs isomers is limited due to potential isomerization under high electron impact energy (31).

In this study, *SIC* fractionation for the removal of *cis*-FAs from mixed fats was evaluated to enhance the determination of VA and EA contents in margarine, butter, cheeses, and ice creams using GCxGC-TOFMS (Table 4). *SIC* treatment increased the the VA contents of TFAs in margarine by 5.8% and EA contents 1.9%. The VA contents of butter and cheese increased by 51.4 and 27.6%, respectively, while EA contents decreased to 38.9 and 44.7%. As a result of *SIC* treatment of ice cream group 1, VA contents increased by 23.9% and EA decreased by 33.6%. In group 2, VA contents increased by 8.4% and EA decreased by 42.0%. Overall, *SIC* treatment resulted in an increase in the proportion of VA in TFA but showed a decrease in EA. Further studies are needed to verify this tendency.

### Characteristics of the V/E ratio in margarine, butter, cheese, and ice cream

As indicated in the previous report (14), the ratio of VA to EA (V/E ratio) is considered an important parameter for evaluating food. In this study, we examined individual TFAs in commonly available market foods such as margarine, butter, cheese and ice cream (25). The proportions of the VA and EA in the margarine, butter, cheese and ice cream, are reported in Table 5. For untreated *SIC*, the V/E ratios were as follows: margarine 1.00, butter 2.94, cheese 3.36, and ice cream 2.02 to 4.03. Based on the reported distribution profile of isomeric *trans*-C18:1, the concentrations of EA and VA in margarine were 23.7 and 13.4%, respectively (32), estimating a V/E ratio of be 0.57. However, for the butter, the concentrations of EA and VA were 1.04 and 4.37%, respectively (16), estimating V/E ratio to be 4.20. Ground beef showed EA and VA concentrations ranging from 0.23 to 0.24% and from 1.09 to 1.13%, respectively (25), estimating V/E ratio to ranging from 4.54 to 4.91. In cheese, EA and VA concentrations ranged from 0.34 to 0.35% and 3.64 to 3.71% (33), respectively estimating the V/E ratios of 10.41 and 10.60, respectively. These proportions align well with those reported in the literature (14), supporting the use of V/E ratios for discriminating of natural and hydrogenated TFAs.

**Table 5:** The V/E ratio of margarine, butter, cheese, and ice creams.

*SIC*	Margarine (*n* = 3)	Butter (*n* = 3)	Cheese (*n* = 7)	Ice cream	
				group 1^a^ (*n* = 11)	group 2^b^ (*n* = 6)
Untreated	1.00 ± 0.03	2.94 ± 0.11	3.36 ± 0.23	2.02 ± 0.33	4.03 ± 0.51
Treated	1.04 ± 0.05	7.28 ± 0.23	7.75 ± 0.21	3.92 ± 0.49	7.53 ± 0.62

a Incorporated hydrogenated fat to milk fat.

b Only natural milk fat.

On the other hand, for treated *SIC*, the V/E ratios were 1.04, 7.28, 7.75 and 3.92–7.53 for margarine, butter, cheese, and ice cream, respectively (Table 5). *Cis*-FAs removal using *SIC* treatment resulted in decreased amounts of *trans*-C18:1 and other isomers and increased VA, particularly 11 t2 and 11 t3, as shown in Figure 2. Thus, accurate estimation of V/E ratio requires the removal of *cis*-FAs from the test samples.

Table 5 presents the V/E ratios for two groups of ice cream treated with *SIC*. Margarine exhibited a V/E ratio of about 1.04, while cheese and butter had ratios of at least 7.2. Ice cream group 1 had a V/E ratio of 3.92, and the ice cream group 2 had a ratio of 7.53. A lower V/E ratio in some ice cream may indicate the addition of the hydrogenated fat. Therefore, ice cream group 1 appears to contain both natural and hydrogenated TFAs. The V/E ratio serves as a valuable tool for deducing the hydrogenated fat content in ice cream. VA, with its demonstrated bioactive properties commonly found in ruminant fats, can help discern between products using N-TFAs and those using H-TFAs or mixtures. The application of this methodology to processed foods enables the determination of added fats as natural TFAs, hydrogenated TFAs, or a combination, allowing for product differentiation.

## Conclusion

In this study, a new method was developed by applying a *SIC* SPE method for *cis*-FAs removing so that accurate measurement of V/E ratios in food could be determined. The difference between V/E ratios applied with and without *SIC* was measured. *Cis*-FAs removal did not affect the analytical ratio of total TFAs, but it did affect the ratio of VA to EA. The accurate V/E ratio suggested in this study could be useful as scientific evidence and basic theory for evaluating new quality parameters of dairy and fatty foods. However, we have not yet precisely confirmed the properties of other TFA isomers excluding EA and VA, and further research is needed.

## Data availability statement

The original contributions presented in the study are included in the article/supplementary material, further inquiries can be directed to the corresponding authors.

## Author contributions

MY: Writing – original draft, Conceptualization, Formal analysis. HL: Methodology, Validation, Writing – original draft, Data curation. K-WL: Supervision, Conceptualization, Funding acquisition, Writing – review & editing. DS: Conceptualization, Funding acquisition, Project administration, Supervision, Writing – original draft, Writing – review & editing.
